# Food-Grade Quercetin-Loaded Nanoemulsion Ameliorates Effects Associated with Parkinson’s Disease and Cancer: Studies Employing a Transgenic *C. elegans* Model and Human Cancer Cell Lines

**DOI:** 10.3390/antiox11071378

**Published:** 2022-07-15

**Authors:** Sabya Sachi Das, Arunabh Sarkar, Siva Chander Chabattula, Priya Ranjan Prasad Verma, Aamir Nazir, Piyush Kumar Gupta, Janne Ruokolainen, Kavindra Kumar Kesari, Sandeep Kumar Singh

**Affiliations:** 1 Department of Pharmaceutical Sciences and Technology, Birla Institute of Technology, Mesra, Ranchi 835215, Jharkhand, India; sabya2049@gmail.com (S.S.D.); prpverma@bitmesra.ac.in (P.R.P.V.); 2 Division of Neuroscience and Ageing Biology, CSIR-Central Drug Research Institute, Lucknow 226031, Uttar Pradesh, India; arunabh.sarkar.bt@gmail.com (A.S.); anazir@cdri.res.in (A.N.); 3 Stem Cell and Molecular Biology Laboratory, Department of Biotechnology, Indian Institute of Technology Madras, Chennai 600036, Tamil Nadu, India; siva.chabathula@gmail.com; 4 Department of Life Sciences, School of Basic Sciences and Research, Sharda University, Plot No. 32-34, Knowledge Park III, Greater Noida 201310, Uttar Pradesh, India; piyush.kumar1@sharda.ac.in; 5 Department of Biotechnology, Graphic Era Deemed to Be University, Dehradun 248002, Uttarakhand, India; 6 Department of Applied Physics, School of Science, Aalto University, 00076 Espoo, Finland; janne.ruokolainen@aalto.fi; 7 Department of Bioproducts and Biosystems, School of Chemical Engineering, Aalto University, 00076 Espoo, Finland

**Keywords:** food-grade quercetin nanoemulsion, *Caenorhabditis elegans*, Parkinson’s disease, neuroprotective effect, chemotherapeutic effect

## Abstract

A nanosized food-grade quercetin-loaded nanoemulsion (QNE) system comprising capmul MCM NF (oil) and cremophor RH 40 (surfactant) was developed using a high-speed homogenization technique. The developed QNE was studied for its significant neuroprotective (anti-Parkinsonism) and cytotoxicity (anticancer) effects against *Caenorhabditis elegans* (*C. elegans*) strains and human cancer cells, respectively. HR-TEM studies revealed that the QNE was spherical with a mean globule size of ~50 nm. Selected area electron diffraction (SAED) studies results demonstrated that QNE was amorphous. In vivo results show that QNE potentially reduced the α-Syn aggregation, increased mitochondrial and fat content, and improved the lifespan in transgenic *C. elegans* strain NL5901. QNE significantly downregulated the reactive oxygen species (ROS) levels in wild-type *C. elegans* strain N2. In vitro results of the MTT assay show that QNE significantly exhibited chemotherapeutic effects in all treated human cancer cells in an order of cytotoxicity: HeLa cells > A549 cells > MIA PaCa-2 cells, based on the IC_50_ values at 24 h. Conclusively, the QNE showed improved solubility, targetability, and neuroprotective effects against the PD-induced *C. elegans* model, and also cytotoxicity against human cancer cells and could be potentially used as an anti-Parkinson’s or anticancer agent.

## 1. Introduction

Dietary flavonoids are the major polyphenolic phytoconstituents that have exhibited numerous promising therapeutic effects in treating neurodegenerative diseases (NDDs), cancer, cardiovascular diseases, microbial infections and many others, mainly due to their free-radical scavenging and anti-oxidative properties [1]. Quercetin (QT) is one of the most essential dietary flavonoids and is primarily found in various herbal sources, including vegetables (onion, broccoli), fruits (apple, blueberry), and grains. Numerous studies have shown that QT exhibits multifaceted therapeutic applications in various NDDs and demonstrates potential pharmacological effects, including anticancer, antimicrobial, antiviral, cardiovascular diseases, and many others [2,3]. QT is one of the most potent natural antioxidants, which exhibits neuroprotective [4] and anticancer effects [5] due to its superior anti-oxidative properties, inhibitory actions against protein kinases, and regulatory action on several gene expressions. The anti-oxidative properties of QT assists in counteracting the excessive formation of ROS, leading to cellular oxidative stress, a pathological condition associated with cancer, and several ageing-mediated neurological diseases [6,7]. Additionally, QT impacts the expression of glutathione S-transferase (a phase II metabolism enzyme), which is recognized as a potent marker for intracellular oxidative stress and influences cell signaling [8]. 

Parkinson’s disease (PD) is the second most common NDD, which progresses slowly and is recognized by the accretion of Lewy neurites/bodies. Both Lewy neurites and Lewy bodies are primarily comprised of accumulated α-synuclein (α-Syn) proteins and post-translationally altered protein by-products. Atypical aggregation of Lewy bodies within the brain is associated with synaptic dysfunctions and the deprivation of dopaminergic neurons of the nigrostriatal dopamine pathway [9]. In addition, the phosphorylation of Serine 129 (S129), a principal constituent of α-Syn, contributes to the accumulation of α-Syn, causing homeostasis and the amalgamation of synaptic vesicle neuronal transmission imperfections [10]. Furthermore, the imbalance between ROS production and the antioxidant defensive system leads to oxidative stress, one of the significant factors in the pathogenesis of numerous diseases and cell ageing processes [11]. Oxidative stress is also presumed to be responsible for protein and gene alterations that are responsible for the hallmarks of various NDDs, including PD [12]. 

Most of the experimental information associated with QT originated either from in vitro or in vivo studies that include complex processes including uptake, metabolism, and interconnections between cells/tissue and organs within an entire animal model, which are sometimes limited [13]. Thus, to overcome these limitations and attain additional information about the biological effects, particularly associated with aging and stress resistance, researchers use *Caenorhabditis elegans* (*C. elegans*), a nematode, as an in vivo model [14]. *C*. *elegans* has a transparent body, exhibits orthologous genes and numerous basic conserved pathways from higher-order animals, and can be effortlessly cultured in an agar medium at room temperature. Moreover, *C*. *elegans* has a shorter lifespan that assists in conducting several biological assays within a limited time frame [15,16]. In addition, the genomic sequence of *C. elegans* and humans highly resembles each other [17]. 

In laboratory experiments, dietary *Escherichia coli* OP50 (*E. coli* OP50) is used as nutrition for *C. elegans*, which is ingested by pumping it within the mouth and directing it within the pharynx before passing into the intestine [18]. The hydrophilic behavior of the growth media and the food behavior (specificity for *E. coli* OP50) of *C. elegans* have restricted their usage as an in vivo model for studying assimilation, absorption, and therapeutic activities, particularly of hydrophobic moieties. However, these limitations could be overcome by establishing emulsion-based delivery systems and a perpetual pumping-in reflex within the pharynx of *C. elegans* [18]. Moreover, QT exhibits poor solubility and low mucosal permeability, leading to lower bioavailability; thus, it becomes necessary to encapsulate QT within a steady nanosystem to increase its bioavailability. To date, numerous nano-carrier-based approaches such as nanoemulsions, polymeric nanoparticles, liposomes, and others have been reported for the encapsulation of QT to achieve improved solubility, permeability, bioavailability, and diseased-site targetability [19].

Despite comprehensive research, the therapeutic possibilities for the management of PD are restricted, offering symptomatic relief only and cannot avoid disease progression completely. Therefore, the present study aimed to establish a food-grade nanoemulsion system for delivering QT into the transgenic *C. elegans* expressing human α-Syn (NL5901 strain) and to evaluate its neuroprotective and pharmacological actions. Also, the potential toxicity of the pure QT and QNE were observed in various carcinogenic cells, including A549 (lung carcinoma epithelial) cells, MIA PaCa-2 (human pancreatic cancer) cells, and HeLa (cervical cancer) cells using the MTT assay method. Moreover, the therapeutic effects of quercetin-loaded nanoemulsion in a transgenic *C. elegans* model have not been reported yet, thus, we believe that these findings would allow the development of novel emulsion-based systems as potential anti-parkinsonism and anticancer agents with improved bioavailability and biocompatibility.

## 2. Materials and Methods

### 2.1. Materials

Quercetin (QT), sodium azide, phosphotungstic acid (PTA, H_3_PW_12_O_40_), 3-(4,5-dimethylthiazol-2-yl)-2,5-diphenyl tetrazolium bromide (MTT), sodium chloride (NaCl), and peptone were purchased from Sigma-Aldrich Chemicals, St. Louis, MO, USA. 2,7-Dichlorodihydrofluorescein diacetate (H2DCFDA) was obtained from Invitrogen, Waltham, MA, USA. Capmul MCM NF (CAP MCM NF) and cremophor RH40 (CR RH 40) were ex gratis from Gattefosse (Saint-Priest, Cedex, France) and BASF (Ludwigshafen, Germany), respectively. Sodium hydroxide (NaOH), doxorubicin hydrochloride (DOX), and organic solvents were procured by Merck, India. Agar was purchased from HIMEDIA, Mumbai, India. Glassware was acquired from Borosil, India. A549 (lung carcinoma epithelial) cells, MIA PaCa-2 (human pancreatic cancer) cells, and HeLa (cervical cancer) cells were purchased from the National Centre for Cell Science, India. These cells were maintained in 10% fetal bovine serum (FBS) comprising Roswell Park Memorial Institute (RPMI) media (Gibco, Waltham, MA, USA) and Dulbecco’s Modified Eagle Medium (DMEM) in a humidified CO_2_ incubator (Thermo Scientific, Waltham, MA, USA) at 37 °C. The cell culture plastics were procured from Corning, Corning, NY, USA. All other chemicals and reagents were of analytical grade and were used as such. 

### 2.2. Formulation of Optimized QNE

The optimized quercetin-loaded nanoemulsion (QNE) was formulated as per our previously reported method [19]. In brief, the nanoemulsion comprised CAP MCM NF (oil) and CR RH 40 (surfactant) was prepared using simple mixing followed by a high-shear homogenization technique. Initially, the drug was solubilized in the oil phase, and then the oil phase was added dropwise to the aqueous phase containing surfactant, followed by constant stirring at 500 rpm using a magnetic stirrer (IKA C-MAG HS 7, Staufen, Germany). QNE was observed for stability and was homogenized at 8000 rpm for 10 min using a high-shear homogenizer (IKA T25, ULTRA TURRAX, Staufen, Germany). Further, the formulation was stored in a sterile environment till further analysis.

### 2.3. Morphological Findings Using HR-TEM Studies

For HR-TEM analysis, the concentrated QNE was diluted using Milli-Q water (1:10). The diluted sample was drop-cast over the carbon-coated copper grid (200 mesh) followed by negative staining using 2% *w/v* phosphotungstic acid (PTA) and was kept for drying at room temperature. Morphological and structural analysis of the optimized QNE was studied using high-resolution transmission electron microscopy (HR-TEM; JEM 2100F, JEOL-200, Peabody, MA, USA) with an accelerating potential of 200 kV. Further, SAED studies were performed to evaluate the physical properties of QNE, either crystalline or amorphous.

### 2.4. Preservation and Culturing of C. elegans Culture

In this study, an *E. coli* strain (OP50) was used by us as a food source for *C. elegans*. The OP50 was seeded appropriately to develop a bacterial lawn over the NGM plates. The growth media for the *C. elegans* culture was developed by adding NaCl (3 g), peptone (2.5 g), and agar (2.5 g) into deionized water (DI) water, and then the solution mixture was autoclaved at 50 °C. After autoclaving, 1 M MgSO_4_ (1 mL), cholesterol (1 mL of 5 mg/mL stock), 1 M KH_2_PO_4_ (25 mL; 6.0), and 1 M CaCl_2_ (1 mL) was added into the solution mixture. Further, the media was gently decanted into Petri plates (90 mm) in a sterile environment and was kept undisturbed till congealed. Once the media was properly congealed, the plates were sterilized under ultraviolet light and were stored at 4 °C till further use. Further, *E. coli*-OP50 (500 μL) was dispensed over the NGM plates using a spreader in a sterile environment. To attain an appropriate bacterial lawn, seeded plates were kept in the incubator at 22 °C overnight. The sub-culturing of *C. elegans* strains was carried out using the pre-seeded NGM plates containing OP50, maintained at 22 °C.

### 2.5. Isolation of C. elegans Embryo

Gravid worms growing over the OP50 seeded NGM plates were harvested using M9 buffer. The worms or nematodes were transferred into a sterilized 15 mL conical tube, centrifuged (1500 rpm; 5 min), and the precipitates collected. The precipitated nematodes (pellets) were resuspended into M9B followed by washing (thrice) to remove any adhered bacteria over the worm surface. Finally, the pellets were treated with an axenizing solution composed of NaOCl (2 mL) and NaOH solution (1 M). The body of nematodes was dissolved by gently mixing the whole solution, causing the discharge of the embryos. The acquired embryos were centrifuged (1500 rpm; 5 min) to achieve pelleted embryos, followed by washing (thrice) using M9 buffer, and then dispersed uniformly over the experimental plates under aseptic conditions.

### 2.6. C. elegans Strains, Staining, and Image Acquisition

In the present study we have used significant strains including, N2: Bristol wild-type strain, NL5901: pkls2386 [unc-54p::alpha-synuclein::YFP + unc-119(+)]. 

The various biological changes in the untreated (control), QT-treated (1 mM), and QNE-treated (1 mM) nematodes were monitored using fluorescent microscopy techniques. Initially, the synchronized worms were washed (thrice) using M9 buffer for the removal of any adhered bacteria from the treatment plates. Then the collected worms were treated with sodium azide (100 mM) for their immobilization. The immobilized nematodes were mounted over clean and sterilized glass slides with the help of coverslips. Lastly, the fluorescence microscope (Carl Zeiss Axio Imager M3 was used for capturing the images of immobilized nematodes.

Moreover, for α-Syn protein aggregation experimentations, *C. elegans* strains NL5901 were used. Further, MitoTracker™ Red CMXRos and Nile Red staining dye were used to enumerate the treated worms’ mitochondrial and fat content respectively. Image acquisition and quantification of images were performed using ZEN2010 image acquiring software and Image J software (Image J, National Institutes of Health, Bethesda, MD, USA). 

### 2.7. C. elegans Lifespan Assay

Age-synchronized worms were obtained by applying the isolated embryos of gravid *C. elegans* onto treated and control plates. After 48 h, ~100 adult worms (L4 stage), observed under a stereo zoom microscope, were selected and relocated onto the freshly arranged feeding plates of both control and treated groups. Thenceforth, daily monitoring and counting of living, dead, and expurgated worms was executed, and during this process, counting was performed by taking two replicate plates for each group. An expurgated set of statistics were documented based on an overall count of mislaid nematodes or the nematodes near the edge of the plates. Further, based on the responses to moderate nudging over the nematodes’ heads, the number of living and dead nematodes was recognized and documented each day. Furthermore, if no response was monitored, the nematode was considered to be dead. Regularly, *C. elegans* were relocated onto freshly arranged treated plates for 20 days and incubated in BOD incubators (22 °C). The grading of worms was executed from the egg phase till its death phase, and Kaplan–Meier survival curves were used for the analysis of the results.

### 2.8. ROS Estimation in C. elegans N2 Strain

The ROS estimation studies were performed in *C. elegans* N2 strain using H_2_DCFDA. The untreated nematodes (control) and treated nematodes were washed using M9 buffer (thrice) and then with phosphate buffer saline (twice). The groups (~100 worms/100 mL media) were individually examined (*n* = 3) and they were added to the experimental wells (OptiPlate-96 F; PerkinElmer), with further addition of 100 mM of H_2_DCFDA (100 μL) into each well. Estimation of the fluorescence intensity (F_int._) from individual wells was noted at three distinct durations, (i): before adding dye (D1), (ii): instantaneously after adding dye (D2), and (iii): one-hour post-incubation after adding dye (D3). The F_int._ was quantified using a multimode plate reader (Perkin Elmer, VICTOR X3) having excitation and emission wavelengths of 485 nm and 520 nm, respectively. The difference in the F_int._ was attained by deducting the values of D1 from D2 and subtracting the obtained value (D2-D1) from the value of D3. The F_int._ of untreated (control), QT-treated (1 mM), and QNE-treated (1 mM) worms were plotted as their mean value. Moreover, statistical significance was attained by Student’s *t*-test (GraphPad Prism software, San Diego, CA, USA). 

### 2.9. Cytotoxicity Study Using MTT Assay

The anticancer activity of pure QT and QNE against selective cancer cell lines (A549, MIA PaCa-2, and HeLa) were analyzed using an MTT assay. In brief, 5 × 10^3^ cells (in each well) were seeded in a 96-wells plate and then were treated with varying concentrations (15 and 500 µg/mL) of different test samples (QT and QNE) under serial dilutions. The study was performed by considering the equivalent weight of the drug (500 µg) in both the test samples (pure QT and QNE), followed by serial dilutions. In addition, cells were also treated with DOX (positive control) with a varying concentration range (1.5 to 100 µg/mL). The treated wells were incubated in a CO_2_ incubator maintained at 37 °C and were observed for 24, 48, and 72 h post-treatment. Later, the percentages of cell viability (% cell viability) for each sample were calculated by calculating the absorbance (Abs.) of samples using the following formula:

$$CV\;\left(\%\right)=\frac{A_{S}-A_{B}}{A_{N}-A_{B}}\times 100$$

where, A_S_ is the Abs. of the sample, A_B_ is Abs. of the blank well and A_N_ is Abs. of the negative control. Further, the IC_50_ values were determined for all the tested samples and the positive and negative controls. The results were signified as mean ± SD and experiments were carried out in triplicate (*n* = 3).

### 2.10. Statistical Analysis

We performed at least two independent experiments for microscopy-mediated quantification assays to treat and study the 100 nematodes for individual groups, as reported in our published literature [20]. However, the F_int._ of nematodes was noted for all sets of slides. The characteristic images within each distinct experiment were randomly captured (*n* = 10). Finally, the quantification of the intensity of each image was performed by Image J analysis software. Further, a one-way ANOVA followed by Tukey’s test was performed to examine the F_int._ for images of each set and ROS values, using GraphPad Prism 5 software; the results are represented as mean ± SD. Furthermore, the *p*-values are signified as * *p* < 0.05, ** *p* < 0.01, *** *p* < 0.001. For all the experiments, adult nematodes at the L4 stage were used. 

Specifically, all the findings for anticancer studies (MTT assay) were analyzed using GraphPad Prism 9.0 and represented as mean ± SD (*n* = 3). The obtained results for pure QT and QNE were examined using multiple *t*-tests and two-way ANOVA for DOX analysis, respectively. Furthermore, the *p*-values are signified as * *p* < 0.05, ** *p* < 0.01, *** *p* < 0.001, **** *p* < 0.0001.

## 3. Results

### 3.1. Formulation and Morphological Assessment

**Figure 1:**
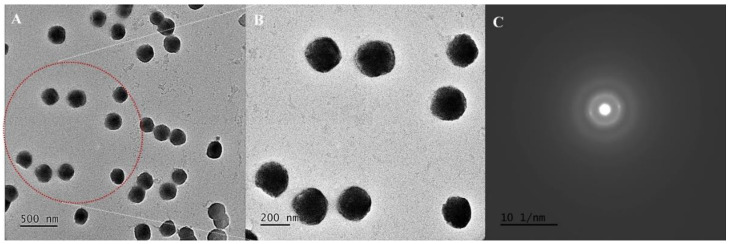
Morphological characterization of optimized QNE: HR-TEM image at 500 nm (**A**) and magnified area (showed with the red dotted area) at 200 nm scale bar (**B**); SAED pattern images (**C**).

The optimized quercetin-loaded nanoemulsion (QNE) was developed as per the method reported in our previously published literature [19]. The optimized QNE comprised of QT (10 mg), CAP MCM NF (250 mg), CR RH 40 (250 mg), with a volume made up to 10 mL using Milli-Q water. Morphological assessments of QNE were performed using HR-TEM. Negative (-ve) staining was performed using PTA (-ve stain) to attain distinct morphological findings. PTA is a typical -ve stain that generally works at a pH range of 6.0 to 8.0 [21]. As per the HR-TEM analysis, QNE were homogeneously distributed, and exhibited stability with a particle size of 50 nm, spherical appearance, and smoother surface (Figure 1A,B). These results are similar to earlier published results [22,23]. Bouchemal et al. reported that parameters including the viscosity of oil, HLB values of surfactants, and miscibility of solvent systems with an aqueous phase play a crucial role in governing the size and morphology of nanoemulsion systems [22]. Moreover, the results of SAED suggested that the QNE exhibited an amorphous nature, showing proper incorporation of the drug within the excipient core system (Figure 1C). Similar findings regarding the effect of excipients on the crystallinity of drug moieties were reported in earlier works from the literature [23,24].

### 3.2. QNE Decreases the α-Synuclein Aggregation in Transgenic C. elegans Strain NL5901

**Figure 2:**
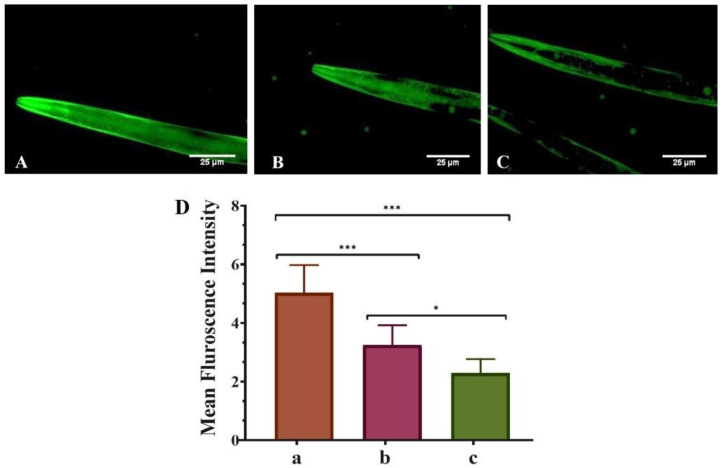
Effect of pure QT and QNE on α-Syn expression in transgenic *C. elegans* NL5901: (**A**) control (OP50 only), (**B**) worms treated with pure QT, (**C**) worms treated with QNE, and (**D**) mean F_int._ for (a) control (OP50 only), (b) worms treated with pure QT, (c) worms treated with QNE; (*n* = 10). One-way ANOVA followed by Tukey’s test was performed for the quantification of graphically represented mean F_int_. (mean ± SD; * *p* < 0.05, *** *p* < 0.001).

This study examined the neuroprotective effects of pure QT and QNE on α-Syn protein aggregation in transgenic *C. elegans* strain NL5901. NL5901 expresses human α-Syn in its muscle cell wall that is tagged with a yellow fluorescent protein. The microscopical findings (Figure 2) demonstrate that the mean F_int._ of untreated worms (only OP50) exhibited the highest level (5.495 ± 0.658) of α-Syn aggregation. However, the mean F_int._ of pure QT-treated worms (OP50 + QT) was less (3.47 ± 0.325) than the untreated worms and least (2.299 ± 0.012) in QNE-treated worms (OP50 + QNE). The results demonstrate that the optimized QNE downregulated the α-Syn expression levels more effectively than the pure QT in the transgenic *C. elegans* expressing human α-Syn (NL5901 strain). These findings suggest that the administration of QNE significantly enhanced the release of QT within the worms, leading to high targetability against α-Syn aggregations with improved bioavailability.

### 3.3. QNE Increases the Mitochondrial Content in Transgenic C. elegans Strain NL5901

Our study investigated the protective role of pure QT and QNE against mitochondrial content using transgenic *C. elegans* strain NL5901 as in vivo model. It was considered that the mean F_int._ was based on mitochondrial content, i.e., high mean F_int._ signifies higher mitochondrial content and vice-versa.

**Figure 3:**
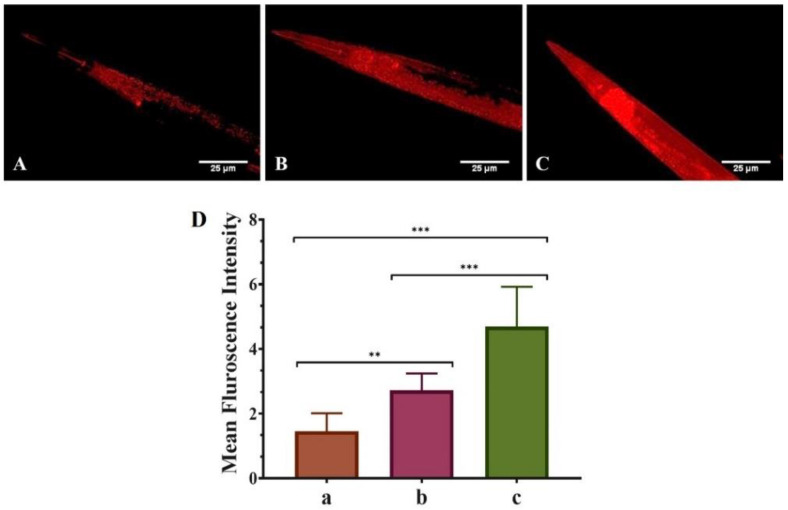
Effect of pure QT and QNE on the mitochondrial changes in transgenic *C. elegans* NL5901: (**A**) control (OP50 only), (**B**) worms treated with pure QT, (**C**) worms treated with QNE, and (**D**) mean F_int._ for (a) control (OP50 only), (b) worms treated with pure QT, (c) worms treated with QNE; (*n* = 10). One-way ANOVA followed by Tukey’s test was performed for the quantification of graphically represented mean F_int_. (mean ± SD; ** *p* < 0.01, *** *p* < 0.001).

Results of the MitoTracker™ Red CMXRos and image acquisition (Figure 3) show that untreated worms (only OP50) exhibited the least mean F_int._ (1.375 ± 0.092), showing poor mitochondrial content. However, the mean F_int._ of pure QT-treated worms (OP50 + QT) was more (2.855 ± 0.544) than the untreated worms and was highest (3.080 ± 1.117) for QNE-treated worms (OP50 + QNE). Thus, the results signify that QNE elevated the overall mitochondrial content in the transgenic *C. elegans* expressing human α-Syn (NL5901 strain) compared with pure QT. 

### 3.4. QNE Increases the Fat Content in Transgenic C. elegans Strain NL5901

**Figure 4:**
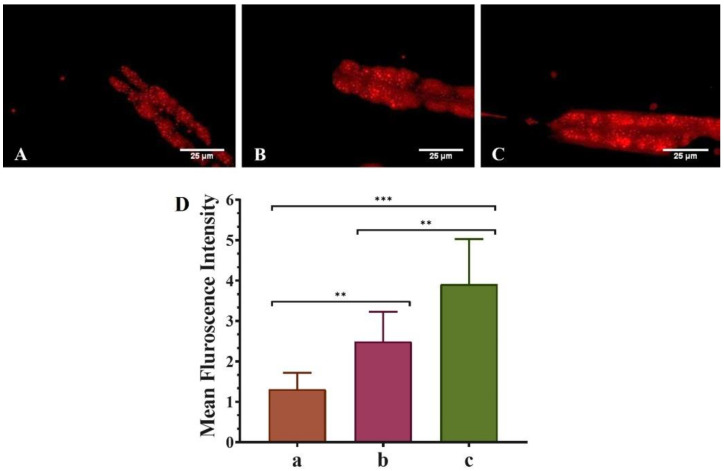
Effect of pure QT and QNE over the fat content in transgenic *C. elegans* NL5901: (**A**) control (OP50 only), (**B**) worms treated with pure QT, (**C**) worms treated with QNE, and (**D**) mean F_int._ for (a) control (OP50 only), (b) worms treated with pure QT, (c) worms treated with QNE; (*n* = 10). One-way ANOVA followed by Tukey’s test was performed for the quantification of graphically represented mean F_int_. (mean ± SD; ** *p* < 0.01, *** *p* < 0.001).

We explored the activity of both pure QT and QNE in regulating the fat content in the transgenic *C. elegans* strain NL5901 (Figure 4). Fat content was detected using Nile red as a staining dye. It was considered that mean F_int._ was based on fat content, i.e., high mean F_int._ signifies higher fat content and vice-versa. The results show that untreated worms (only OP50) exhibited the smallest mean F_int._ (1.44 ± 0.014), showing poor fat content. However, the mean F_int._ of pure QT-treated worms (OP50 + QT) was more (2.64 ± 0.113) than the untreated worms and was highest (4.595 ± 0.516) for QNE-treated worms (OP50 + QNE). Thus, the results signify that QNE elevated the overall fat content in transgenic *C. elegans* expressing human α-Syn (NL5901 strain).

### 3.5. QNE Increases the Longevity in C. elegans NL5901 Strain and Downregulates the ROS Levels in Wild-Type C. elegans N2 Strain

**Figure 5:**
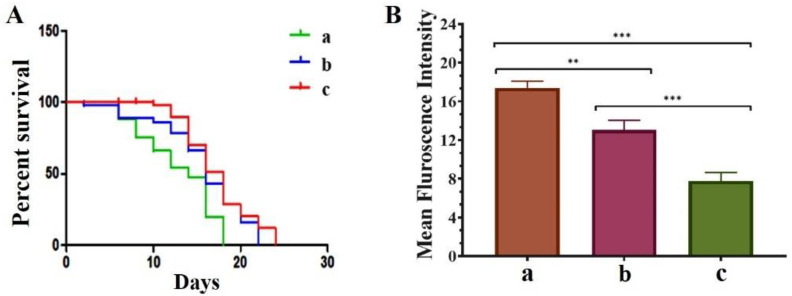
Effect of pure QT and QNE on: (**A**) percent survival of treated transgenic *C. elegans* NL5901 (a) control (NL5901 only), (b) worms treated with pure QT, and (c) worms treated with QNE; and (**B**) mean F_int._ of ROS level in *C. elegans* N2 for (a) control (OP50 only), (b) worms treated with pure QT, and (c) worms treated with QNE; (*n* = 10). One-way ANOVA followed by Tukey’s test was performed for the quantification of graphically represented mean F_int_. (mean ± SD; ** *p* < 0.01, *** *p* < 0.001).

It has been reported that treatment with QT efficiently prolonged the lifespan, inhibited age-associated motility delay, induced heat-stress tolerance, and reduced the production of intercellular and mitochondrial ROS in *C. elegans* strains [15]. Thus, we explored the effect of pure QT and QNE on the longevity of the treated *C. elegans* NL5901 strain. We observed that treatment with QNE significantly increased the longevity up to 24 days, whereas in QT-treated worms, the longevity was increased up to 22 days. In untreated *C. elegans* (control group) the lifespan was found to be up to 18 days (Figure 5A). 

We further explored the effects of pure QT and QNE on the overall ROS content in wild-type *C. elegans* N2 strain. In our study, the estimation of ROS was performed using H_2_DCFDA. It was expected that the treatment samples would exhibit ROS scavenging properties. The results of the ROS analysis show that the QNE (7.78 mean intensity) decreased the overall ROS content more efficiently than pure QT (13.06 mean intensity) in wild-type *C. elegans* (Figure 5B). Grunz and co-workers stated that QT (100 M; 48 h), a potent natural antioxidant, decreased levels of mitochondrial ROS in the *C. elegans* in vivo model by about 70% and also improved the lifespan (18.4%) [25]. 

### 3.6. QNE Potentially Reduced the Cell Viability of Treated Human Cancer Cells

**Figure 6:**
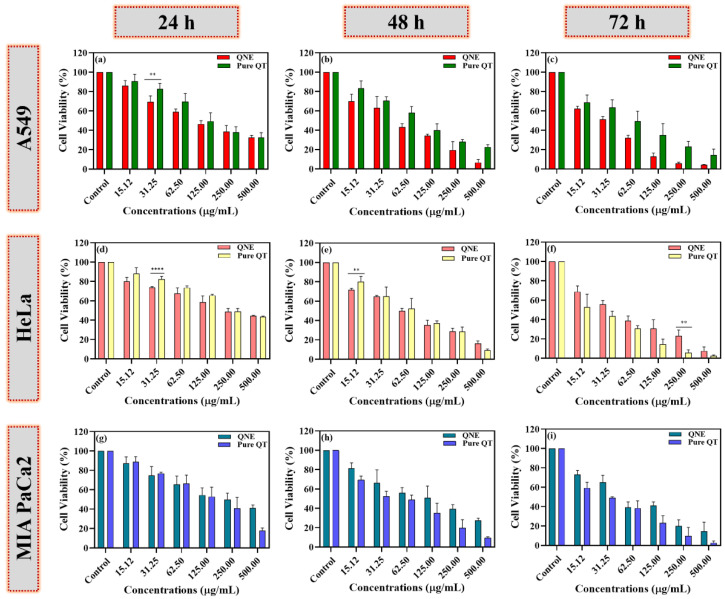
The percentages of cell viability of free QT- and QNE-treated cells were calculated in a dose- and time-dependent manner using the MTT test on different human cancer cells: A549 (**a**–**c**), HeLa (**d**–**f**), and MIA PaCa-2 cells (**g**–**i**). The data were analyzed using GraphPad Prism 9.0 software and represented as mean ± SD (*n* = 3). The obtained results for pure QT and QNE were further examined using multiple *t*-tests. The *p*-values are signified as ** *p* < 0.01, **** *p* < 0.0001.

The anticancer activity of free QT, QNE, and positive control (DOX) was analyzed using the MTT test on different cancer cell lines, A549, MIA PaCa-2, and HeLa. The IC_50_ values for all samples and the positive control (DOX) were calculated at 24 h (Figure 6).

However, the presence of surfactant and oil in QNE could have increased the cytotoxic potential of pure QT against the treated human cancer cells. The chemotherapeutic potential of QT is mainly due to its ability to promote cell viability loss, apoptosis, and autophagy through the modulation of various molecular pathways associated with cancer progression [26]. In our study, QNE showed potential cytotoxicity against treated HeLa cells (Figure 6a), A549 cells (Figure 6d), and MIA PaCa-2 cells (Figure 6g), with IC_50_ values of 34.25 ± 1.213 µg/mL, 40.33 ± 6.221 µg/mL, and 43.58 ± 9.160 µg/mL, respectively, at 24 hr. In addition, the pure QT-treated cells also exhibited potential chemotherapeutic activity against treated HeLa cells (Figure 6a), A549 cells (Figure 6d), and MIA PaCa-2 cells (Figure 6g), with IC_50_ values of 63.23± 1.884, 63.46 ± 8.485, and 84.14 ± 8.607, respectively, at 24 h. The IC_50_ values of pure QT-treated carcinogenic cells were found to be more than QNE-treated carcinogenic cells, signifying QNE exhibits superior activity at 24 h treatment. Moreover, similar findings were observed in earlier studies for HeLa cells [27,28], A549 cells [29,30], and MIA PaCa-2 cells [31,32]. Moreover, there were no significant differences between the pure QT and QNE against carcinogenic cells, except for few concentrations including: A549 (31.25 µg/mL; *p* < 0.01), HeLa cells (31.25 µg/mL; *p* < 0.0001) at 24 h; HeLa cells (15.12 µg/mL; *p* < 0.01) at 48 h; and HeLa cells (250 µg/mL; *p* < 0.01) at 72 h of treatment. This shows that QNE exhibited superior activity against HeLa cells and A549 cells at a lower concentration.

Overall, the results show that both QNE and pure QT exhibited cytotoxic effects against all the treated cancer cell lines in a dose- and time-dependent manner. QNE was found to be more toxic to cervical cancer (HeLa) cells, followed by lung (A549) and pancreatic (MIA PaCa-2) cancer cells (Figure 6). Moreover, DOX exhibited potent anticancer activity on all three tested human cancer cells in a dose- and time-dependent manner, indicating that cancer cells were not resistant (Figure S1).

## 4. Discussion

Quercetin (QT), a natural flavonoid and anti-oxidant, has exhibited potential neuroprotective (anti-parkinsonism) [4] and chemotherapeutic (anticancer) [5] effects. QT potentially inhibits intracellular and mitochondrial ROS, leading to reduced oxidative stress in neuronal cells [1]. Unfortunately, QT, whose neuroprotective and chemotherapeutic effects have been established in various studies, shows limitations due to its poor aqueous solubility and low oral bioavailability, which could be overcome through nanotechnological approaches [3]. Moreover, despite extensive research studies about PD, no potential medications have been reported with high therapeutic efficacy. 

Here, we explored the neuroprotective and chemotherapeutic potential of the natural antioxidant (QT)-encapsulated nanoemulsion (QNE) system against *C. elegans* strains and human cancer cells, respectively. One of the essential facts about using food-grade excipients in QNE fabrication is that although QT is highly soluble in organic solvents like ethanol and DMSO, such solvents’ usage produces adverse effects in *C. elegans* when administered directly [33]. Morphological findings demonstrated that the QNE exhibited a smoother and spherical appearance due to the proper blending of drugs within the excipient system [23]. This improved the solubility and stability of the drug, leading to improved bioavailability.

The progression of PD has been reported to be slow and is mainly recognized by the deposition of Lewy neurites/bodies, primarily comprised of accumulated α-Syn proteins. Here, we observed that QNE significantly downregulated α-Syn aggregations in the transgenic *C. elegans* expressing human α-Syn (NL5901 strain). This could be possibly due to the ability of QT to bind with α-Syn specifically through covalent bonds, resulting in the α-Syn–QT complex [34]. In an earlier study, the neuroprotective role of QT against synucleinopathies by efficiently hindering the aggregations of α-Syn has been reported [34]. In another study, researchers demonstrated that QT exhibits neuroprotective effects in SH-SY5Y (neuroblastoma-derived) cells against rotenone-induced neuronal injuries by modulating the process of autophagy [35]. 

Mitochondria exhibit a vital role in smooth neuronal functioning; however, loss of mitochondrial content and mitochondrial dysfunction has been reported as significant factor causing the neurodegeneration detected in PD. QT has been found to have a protective effect against impaired mitochondrial biosynthesis contributing to mitochondrial dysfunction in PD [36]. The results were identical to the previously published literature where the researchers explored the neuroprotective effects of QT in 6-hydroxydopamine(6-HD)-induced rats in vivo and 6-HD-treated PC12 (derived from rat pheochromocytoma) cells in vitro. These in vitro studies showed that QT (20 μM)-treated PC12 cells exhibited improved mitochondrial functions, decreased oxidative stress and α-Syn aggregations, and enhanced mitophagy marker levels. The in vivo findings revealed that QT inhibited neuronal death, mitochondrial damage, and α-Syn aggregation in PD rat models [37]. Moreover, higher intakes of cholesterol and polyunsaturated fatty acids (PUFA), particularly monounsaturated fatty acids (MUFA), might reduce the risk of PD in both men and women. Moreover, the FA are the primary constituents present in neuronal cell membranes and are vital for upholding their structural integrity and neurological functions [38,39]. Additionally, PUFAs play a critical role in maintaining the fluidity and permeability of the neuronal membrane, triggering phospholipases, reprocessing synaptic vesicles, and inhibiting ion channels [40]. Here, QNE significantly improved both mitochondrial and fat content in the treated transgenic *C. elegans* expressing human α-Syn (NL5901 strain). 

Lifespan is a practical and supportive physiological factor that helps in assessing the biological effects of a compound in various animal models, either positive (increased longevity or non-toxic) or negative (reduced longevity or toxic) [41]. QNE potentially increased the longevity of the treated transgenic *C. elegans* expressing human α-Syn (NL5901 strain). Similar activities have been reported in earlier studies, where QT potentially prolonged the lifespan and increased stress tolerance in *C*. *elegans* [42,43,44].

Furthermore, mitochondria are susceptible to oxidative stress (majorly instigated due to ROS) and oxidative stress is responsible for the distraction of mitochondrial electron transport chains leading to reduced energy production by mitochondria. Thus, it becomes necessary to inactivate ROS using antioxidants and associated mechanisms [45]. In our study, QNE potentially downregulated the ROS level in the wild-type *C. elegans* N2 strain that could be responsible for neutralizing oxidative stress within the nematode. The mechanism behind the anti-oxidative effects of QT is due to the reaction of QT with free radicals, leading to the formation of a stable moiety with less reactivity, thus preserving the viability of cells [7].

In addition, the cytotoxic potential of QT has been well known, however, biological limitations of QT have restricted its direct usage [3]. Here, in our study, QT-encapsulated nanoemulsions exhibited potential cytotoxic effects against all the treated human cancer cells, including HeLa (cervical cancer) cells, A549 (lung carcinoma epithelial) cells, and MIA PaCa-2 (human pancreatic cancer) cells. El-Gogagry and co-workers reported that the cell viability of QT (10 μM)-treated cell lines after 48 h of treatment was found to be 81.70% ± 5.75 (C6 rat glioma cells), 56.09% ± 8.20 (B16F10 murine melanoma cells), 47.08% ± 8.73 (CT26 murine colon cancer cells), and 43.99% ± 3.95 (HeLa cells) [27]. In another study, QT-treated HeLa cells exhibited a significant reduction at 100 µM (24 h). In a study, QNE exhibited cytotoxicity against A549 cells (IC_50_: 300 μg/mL; 48 h) in a dose- and time-dependent manner [29]. In another study, MTT assay findings stated that QT significantly decreased the cell viability of A549 cells (IC_50_: 52.35 ± 2.44 μM) and MCF-7 breast cancer cells (IC_50_: 41.78 ± 2.21 μM) in a dose-dependent manner [30]. Moreover, QT exhibited no toxicity against treated lymphocytes (similar dose), and thus, QT was found to be safe in non-cancer cells, explicitly targeting carcinogenic cells [28]. An earlier study demonstrated that QT (0–75 μM) reduced the cell numbers of both cells, including MIA PaCa-2 and BxPC-3 (temperately differentiated cells) after 48 h of treatment, with the smallest QT concentration of 10 μM [31]. In another study, QT intensely decreased cell counts, enhanced autophagy, and induced apoptosis in a dose-dependent manner in gemcitabine (GEM)-resistant MIA Paca-2 cells [32]. 

## 5. Conclusions

Taken together, we developed food-grade QT-loaded nanoemulsion (QNE) using a high-speed homogenization technique with improved solubility and oral bioavailability of quercetin. QNE were spherical with a globule size of ~50 nm (TEM studies) and were amorphous, signifying a complete blend of QT within the excipient system. Moreover, QNE decreased the α-synuclein expression, increased the mitochondrial and fat content, and improved the longevity in transgenic *C. elegans* strain NL5901. In addition, QNE downregulated the ROS levels in wild-type *C. elegans* strain N2. Furthermore, QNE exhibited superior cytotoxicity against all the treated cancer cell lines with an order of cytotoxicity: HeLa cells > A549 > MIA PaCa-2 cells. Also, DOX (positive control) exhibited potent anticancer activity on all three tested cells indicating that the cancer cells were not resistant. These findings could be used to set nanoplatforms encapsulating potential natural antioxidants with improved neuroprotective effects against neurological diseases like Parkinson’s disease and also for targeting diverse cancer types. Moreover, findings at the pre-clinical and clinical levels could put forward a precise mechanism associated with quercetin nanocarriers in neurological diseases and cancer therapy.

## Data Availability

Data is contained within the article and supplementary materials.

## Supplementary Materials

The following supporting information can be downloaded at: https://www.mdpi.com/article/10.3390/antiox11071378/s1, Figure S1: The percentage of cell viability with DOX was calculated in a dose- and time-dependent manner using the MTT test on different human cancer cells: A549 (a), HeLa (b), and MIA PaCa-2 cells (c). The data were analyzed using GraphPad Prism 9.0 software and represented as mean ± SD (*n* = 3). The obtained results for DOX were further examined using a two-way ANOVA analysis. The *p*-values were signified as * *p* < 0.05, ** *p* < 0.01, *** *p* < 0.001, **** *p* < 0.0001.
