# Supplementary material for: Food-Grade Quercetin-Loaded Nanoemulsion Ameliorates Effects Associated with Parkinson’s Disease and Cancer: Studies Employing a Transgenic C. elegans Model and Human Cancer Cell Lines
Source: Antioxidants (Basel). 2022 Jul 15;11(7):1378. doi: 10.3390/antiox11071378 (PMC9312062; doi:10.3390/antiox11071378)
Supplement: Supplementary file 1 [file antioxidants-11-01378-s001.zip › antioxidants-1802852-supplementary.pdf]

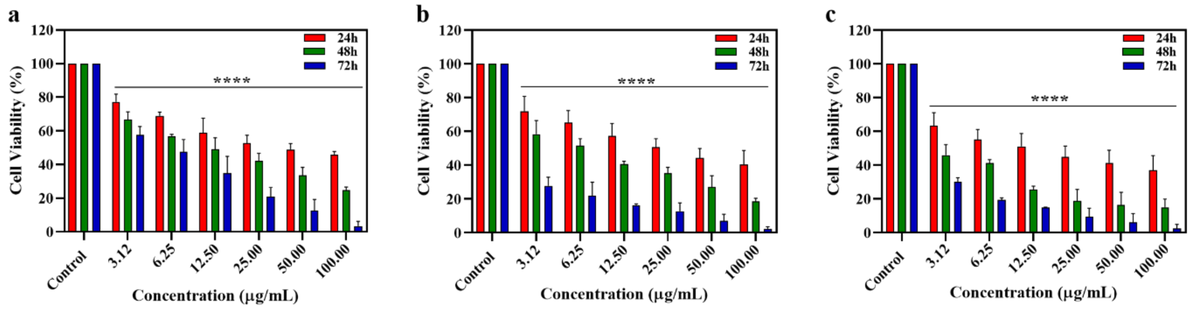


**Figure S1.** The percentage of cell viability of DOX were calculated in a dose-and time-dependent manner using MTT test on different human cancer cells: A549 (a), HeLa (b), and MIA PaCa-2 cells (c). The data were analyzed using GraphPad Prism 9.0 software and represented as mean ± SD (n = 3). The obtained results for DOX were further examined using two-way ANOVA analysis. The p-value were signified as $^{*}p < 0.05$, $^{**}p < 0.01$, $^{***}p < 0.001$, $^{****}p < 0.0001$.
